# Transcriptome and single-cell analysis reveal disulfidptosis-related modification patterns of tumor microenvironment and prognosis in osteosarcoma

**DOI:** 10.1038/s41598-024-59243-9

**Published:** 2024-04-22

**Authors:** Linbang Wang, Yu Liu, Jiaojiao Tai, Xinyu Dou, Hongjuan Yang, Qiaochu Li, Jingkun Liu, Ziqiang Yan, Xiaoguang Liu

**Affiliations:** 1 Department of Orthopaedics, Peking University Third Hospital, Beijing, People’s Republic of China; 2 Department of Orthopedics, Honghui Hospital, Xi’an Jiaotong University, No. 555, Youyi Road, Beilin District, Xi’an, 710054 Shaanxi People’s Republic of China; 3 School of Foreign Studies, Xi’an Medical University, Xi’an, 710054 Shaanxi People’s Republic of China; 4 Department of Orthopedic Surgery, The First Affiliated Hospital, Chongqing Medical University, Chongqing, 400016 People’s Republic of China

**Keywords:** Disulfidptosis, Osteosarcoma, Single-cell analysis, ACTB, HMGB1, Bone cancer, Cancer microenvironment, Tumour biomarkers

## Abstract

Osteosarcoma (OS) is the most common malignant bone tumor with high pathological heterogeneity. Our study aimed to investigate disulfidptosis-related modification patterns in OS and their relationship with survival outcomes in patients with OS. We analyzed the single-cell-level expression profiles of disulfidptosis-related genes (DSRGs) in both OS microenvironment and OS subclusters, and HMGB1 was found to be crucial for intercellular regulation of OS disulfidptosis. Next, we explored the molecular clusters of OS based on DSRGs and related immune cell infiltration using transcriptome data. Subsequently, the hub genes of disulfidptosis in OS were screened by applying multiple machine models. In vitro and patient experiments validated our results. Three main disulfidptosis-related molecular clusters were defined in OS, and immune infiltration analysis suggested high immune heterogeneity between distinct clusters. The in vitro experiment confirmed decreased cell viability of OS after ACTB silencing and higher expression of ACTB in patients with lower immune scores. Our study systematically revealed the underlying relationship between disulfidptosis and OS at the single-cell level, identified disulfidptosis-related subtypes, and revealed the potential role of ACTB expression in OS disulfidptosis.

## Introduction

OS is one of the most prevalent forms of malignant bone cancer and is primarily detected in teenagers and young adults^1^. OS has poor prognosis owing to its high recurrence rate and distant metastatic features^2,3^. Many advanced treatment strategies have been applied for OS, such as chemotherapy, surgery, amputation, and immunotherapy. However, the 5-year survival rate of patients with OS, which is approximately 60–70%, has not significantly improved over the past 50 years^4,5^. Owing to the high genetic heterogeneity of OS cells and lack of reliable and accurate biomarkers, a considerable number of patients with OS already have metastases upon diagnosis, which has become a great challenge for OS management^6^. Therefore, the molecular mechanisms underlying OS progression and reliable prognostic OS biomarkers require further investigation.

Programmed cell death, including apoptosis, autophagy, pyroptosis, ferroptosis, necroptosis, and others^7^, is known as an essential physiological and pathological processes to remove damaged cells for tissue homeostasis^8–10^. Recently, a novel cell death pathway known as disulfidptosis was reported by Liu et al.^11^, which is defined as rapid cell death caused by the abnormal accumulation of disulfide in SLC7A11 hyperexpressing cells and induced disulfide stress under glucose starvation conditions. Previous studies have shown that the abnormal accumulation of cystine and other disulfide compounds in cells induces disulfide stress and is highly toxic to cells^11^. The reduced form of nicotinamide adenine dinucleotide phosphate (NADPH) provides key reducing power to counteract disulfide bond stress and maintain cell survival^12,13^. Cytoplasmic NADPH pools are mainly produced from glucose via the pentose phosphate pathway. In cancer cells with abnormal SLC7A11 expression, cystine uptake is high, and the reduction of cystine to cysteine, when combined with glucose starvation, depletes the NADPH pool, leading to a massive accumulation of intracellular disulfide molecules and rapid cell death^12,13^. However, the mechanism underlying cell death in OS remains unclear.

Previous studies have shown that SLC7A11 is relatively overexpressed in OS cells^14^, and its high expression level is positively correlated with the proliferation and invasion of OS cells^15^; therefore, we investigated the role of DSRGs in predicting the prognosis of OS. In this study, transcriptome expression profiles downloaded from public databases, related clinical parameters, and single-cell transcriptome data of OS were analyzed. We first constructed a landscape of DSRGs expression features at both the microenvironment and tumor subcluster levels by applying single-cell analysis and further established and validated a prognostic model based on DSRGs. Next, we identified the exogenous cytokine HMGB1, which may induce disulfidptosis, using further intercellular communication analysis. This study provides a novel approach for prognostic prediction of OS. In addition, we screened the key gene, *ACTB*, in the disulfidptosis process of OS cells and performed in vitro experiments to test its reliability as a biomarker in patient samples.

## Materials and methods

### Data acquisition

Single-cell RNA-sequencing data were obtained from the GEO database https://www.ncbi.nlm.nih.gov/geo/query/acc.cgi?acc=GSE152048. OS transcriptome sequencing data were downloaded from the Cancer Genome Atlas (TARGETs) https://ocg.cancer.gov/programs/target.

### Single-cell transcriptome analysis

The scatter R package was used for quality control of single-cell RNA-seq data^16^. The scimpute R and scran R packages were used for imputation and normalization, respectively. Anchors were then determined using the FindIntegrationAnchors function and passed to the Integrate Data function to form a Seurat object for downstream analysis. The subtypes of OS cells and other cell types in the OS microenvironment were individually identified using SingleR^17^. AUCell scores were applied to analyze the different biological activities of cell clusters, and the org.Hs.eg.db R package enrichplot was used for functional analysis. The Monocle2 (version 2.4.0) package was used for single-cell pseudo-time trajectory construction and identification of gene expression changes during cell differentiation. Intercellular interactions between OS cells and other cell types in the tumor microenvironment were investigated using NicheNet^18^.

### Unsupervised clustering of OS samples

Initially, a total of 24 DSRGs were obtained according to the previous report by Liu et al.^11^. Based on 24 DSRGs expression profiles, we used the unsupervised clustering analysis (“ConsensusClusterPlus” R package, version 2.60) and classified the 98 OS samples into subclusters by applying the k-means algorithm with 1000 iterations. The maximum subtype number k (k = 9) was chosen and the optimal cluster number was evaluated based on the CDF curve and a consistent cluster score (> 0.9).

### GO, KEGG, and single-sample gene set enrichment analyses

Functional enrichment analyses of GO and KEGG pathways were conducted using the clusterProfiler R package (version 3.14.3) "to determine enriched biological processes (BPs), molecular functions (MFs), and cellular components (CCs); terms or pathways with P < 0.05 were regarded as statistically enriched. The GSVA and GSEA Base R packages were used to obtain the enrichment scores for hub gene functions in the OS transcriptome data.

### Construction of predictive model based on multiple machine learning methods

The “caret” R packages (version 6.0.91) were used to establish machine learning models including RF and SVM. RF is a machine-learning approach that uses various independent decision trees for classification or regression prediction^19^. The SVM algorithm generates a hyperplane in a characteristic space to identify positive and negative instances^20^. The LASSO Cox regression model (R package “glmnet”) was then utilized to narrow down the candidate genes and to develop the prognostic model. A nomogram model was established using the “rms” R package (version 6.2.0). Calibration curve and DCA were used to estimate the predictive power of the nomogram. The ROC curve analysis was performed to verify the diagnostic value of the diagnostic model.

### Immune cell infiltration and correlation analysis with DSRGs

The CIBERSORT algorithm (http://cibersort.stanford.edu/) was applied to estimate the of 22 types of immune cells in different groups on the gene expression data. Cell types with P-values < 0.05 were considered to be differentially abundant immune cell fractions. To further demonstrate the association between DSRGs and OS-related immune cell properties, we analyzed the correlation coefficients between DSRGs expression and the relative percentage of immune cells. Spearman correlation coefficients with P-values below 0.05 represented a significant correlation. Finally, the results were exhibited using the “corrplot” R package (version 0.92).

### Drug sensitivity analysis

The R pRRophetic packages were applied to perform drug sensitivity analysis to screen potential therapeutic drugs for OS; P < 0.05 was set as the screening criterion.

### In vitro experiment

OS cell line 143B was authenticated and tested for mycoplasmas (ATCC, Manassas, VA, USA). The 143B cell line was grown in complete DMEM medium (Gibco, Gaithersburg, MD) with 10% FBS (Gibco, Gaithersburg, MD), 100 U/mL penicillin, and 100 μg/mL streptomycin (Invitrogen, Carlsbad, CA). Cells were cultured in 5% CO_2_ humidified incubator at 37 °C and treated with 300 ng/mL HMGB1 [recombinant HMGB1 protein, Abcam, cat. no. ab167718] in the HMGB1 group.

### siACTB RNA interference

Cells at an appropriate density were incubated for 24 h. ACTB siRNA was transfected with Lipo3000 (31985070, Thermo Fisher Scientific, Waltham, MA, USA) in OPTI-MEM according to the manufacturer’s instructions. The supernatants were discarded 24 h later, and fresh medium was added Cells were harvested after 72 h for further experiments. The siRNA sequences used were as follows:

siACTB-specific siRNA:

- 5′-UAAUGUCACGCACGAUUUCCC-3′,

Non-sense siRNA:

- 5′-UUCUCCGAACGUGUCACGUTT-3′.

### Cell viability assay

Cells were precisely inoculated into 24-well culture plates, and cells with 3 Wells were taken out of each group at fixed intervals for counting and calculating the mean value. The cell growth curve was described with culture time as the horizontal axis and cell number as the vertical axis.

### Patients

OS tissues were surgically resected from nine patients at Honghui Hospital between January 2023 and March 2023. The inclusion criteria were a pathological diagnosis of OS and resection. The exclusion criteria were patients with metastasis, recurrence, and incomplete clinical data. Adjacent tissue samples (n = 9) were collected from the same patient. Informed consent was obtained from all patients in this study. This study was approved by the Ethics Committee of Honghui Hospital and was conducted in accordance with the Declaration of Helsinki (Approval Number: 202303051).

### qRT-PCR

RNA was isolated from cultured OS cells and human tissues using a UNIQ column RNA Extraction Kit (Sangon Biotech, China). Reverse transcription was performed using the RR047 cDNA Synthesis Kit (TaKaRa, China). qRT-PCR was conducted in a 7500 Real-Time PCR System (Applied Biosystems, Foster City, CA) using the 2× Power SYBR^®^ Green PCR Master Mix (Invitrogen, USA). Gene expression levels were normalized to *GAPDH* expression. The Wilcoxon test was used for comparisons between two groups. Statistical significance was set at P < 0.05. The following primer sequences were used:

- *ACTB*-F (5′-GTGCTATCCCTGTACGCCTC-3′),
- *ACTB*-R (5′-AATGCCAGGGTACATGGTGG-3′),
- *GAPDH*-F (5′-GCTGCTCTTGGCTCTCAACT-3′), and
- *GAPDH*-R (5′-GGCATAGGGCTGGTAATGCT-3′).

### Western blotting

The protein expression level of ACTB is quantied by comparing with GAPDH. Treated cells of different group were washed with phosphate-buffered saline and homogenized in 300 μL of radio immunoprecipitation assay buffer supplemented with protease inhibitors and phosphatase inhibitors and then centrifuged at 18,000*g* for 10 min at 4 °C. Protein concentration was determined by BCA assay (Pierce, Rockford, IL, USA) using bovine serum albumin as the standard. Proteins (20 μg/lane) were separated by sodium dodecyl sulfate polyacrylamide gel electrophoresis (SDS-PAGE) and transferred to nitrocellulose membranes (Osmonics, Minnetonka, MN, USA). The membranes were incubated with specific primary antigen (ACTB, Affinity, cat no: AF7018, GAPDH, Affinity, cat no: AF7021) in tris-buffered saline (TBS) containing 0.05% Tween-20 (TBS-T) and 5% non-fat dry milk at 4 °C overnight. After three washes with TBS-T, the blots were incubated with peroxidase-conjugated IgG for 1 h at room temperature, visualized using enhanced chemiluminescence (Amersham Biosciences, Piscataway, NJ, USA), photographed on a ChemiDoc system, and analyzed using the Image Lab Software (Bio-Rad Laboratories, Hercules, CA, USA).

### Informed consent

Informed consent was obtained from all subjects involved in the study.

## Results

### Overall profile of DSRGs expression in cell components of the OS tumor microenvironment (TME)

**Figure 1:**
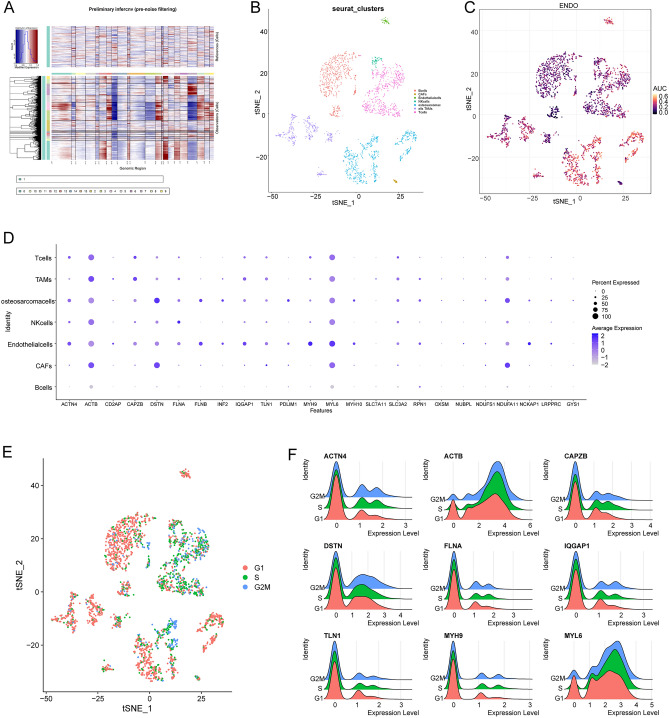
Overall profile of DSRG expressions in cell components of osteosarcoma tumor microenvironment. (**A**) The hierarchical heatmap illustrating CNVs in lesions from the osteosarcoma sample. (**B**) The t-SNE plot showing the seven cell clusters including one malignant cluster from the osteosarcoma sample. (**C**) Scatter plot showing the overall expression feature of DSRGs in each cell cluster. (**D**) Dotplots showing the 24 DSRGs expression across the seven cellular clusters, where the size of dots represents the proportion of cells expressing the genes and the color spectrum indicates the mean expression levels of the genes. (**E**) Scatter plot showing the recognized cell cycle of each cell. (**F**) The ridge plot illustrating the correlation between DSRGs expression and the cell cycle, the red one represent the cell amount in G1 phase, the green one represent the cell amount in S phase, the blue one represent the cell amount in G2M phase.

A scRNA-seq analysis of OS single-cell transcriptome data was implemented to reveal the landscape of cellular diversity. The quality control assessment was carried out firstly (Fig. S1), where cells were initially clustered into 16 major clusters. Chromosomal copy number variation (CNV) was subsequently calculated by inferCNV to identify malignant cells^21^ (Fig. 1A). Seven main segregated cell clusters were next identified (Fig. 1B) using t-distributed stochastic neighbor embedding (t-SNE) analyses. The cell type of each cluster were identified by singleR according to expression profiles^17^, including OS cells, B cells, cancer-associated fibroblasts (CAFs), endothelial cells, NK cells, tumor-associated macrophages (TAMs), and T cells. In order to further observe the disulfidptosis level of the osteosarcoma, profiles of DSRGs expression among the cellular clusters were illustrated by scatter plot (Fig. 1C) and dot plots (Fig. 1D). OS cells, endothelial cells, and TAMs had higher overall DSRGs expression, and particularly, the expression of *ACTB, DSTN, MYL6*, and *NDUFA11* among all DSRGs was relatively high in most cell clusters. Moreover, *DSTN, PDLIM1, MYH10*, and *NDUFA11* were relatively overexpressed in OS cells. *FLNA* were relatively overexpressed in NK cells. *MYH9, NCKAP1*, and *MYL6* were relatively over expressed in endothelial cells. *ACTB* and *DSTN* were relatively overexpressed in CAFs (Fig. 1D). The results of the cell cycle-related expression analysis showed that the expression of *ACTB* and *MYL6* in G1 phase cells was relatively low, but was high in G2M and S phases (Fig. 1E,F), which suggests that disulfidptosis of OS may be potentially related with the expression of the cell cycle genes.

### Identification of disulfidptosis clusters in OS with their functional enrichment and immune infiltration characteristics

**Figure 2:**
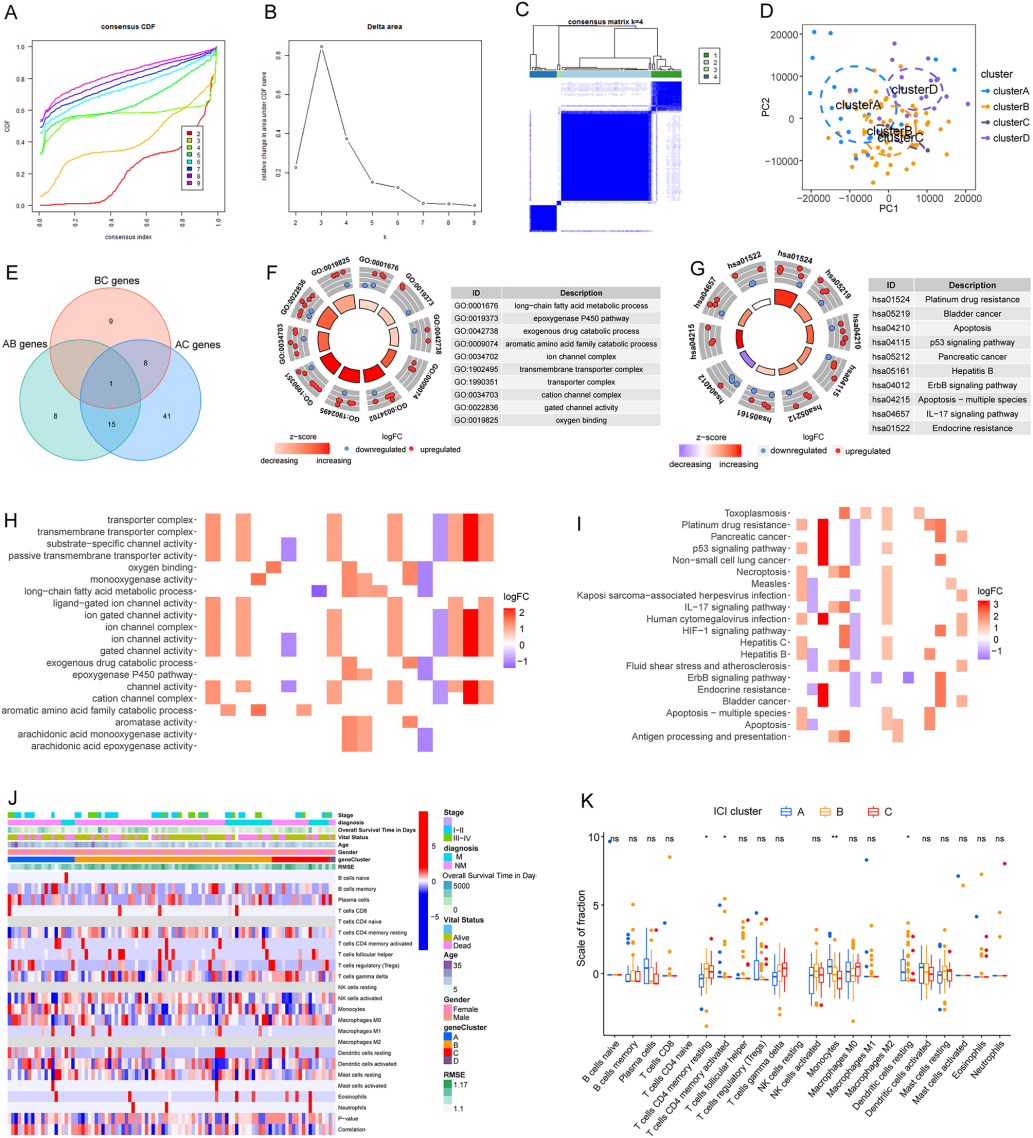
Identification of disulfidptosis-related molecular clusters in OS. (**A**) Representative cumulative distribution function (CDF) curves for sample clustering. (**B**) CDF delta area curves. (**C**) Consensus clustering matrix when k = 4. (**D**) PCA showing the distribution of four subtypes. (**E**) The intersection of differentially expressed genes among three main disulfidptosis-related molecular clusters. (**F**) Circle plot of GO pathway activities of the intersections genes, the shade of the color shows the increasing of the z-score, the blue dot illustrate the downregulated ones and the red dot illustrate the downregulated ones. (**G**) Circle plot of KEGG pathway activities of the intersections genes, the shade of the color shows the increasing of the z-score, the blue dot illustrate the downregulated ones and the red dot illustrate the downregulated ones. (**H**) Heatmap of GO pathway activities of the intersections genes, the shade of the color shows the increasing of the logFC. (**I**) Heatmap of KEGG pathway activities of the intersections genes, the shade of the color shows the increasing of the logFC. (**J**) Identification of molecular and immune characteristics among the three disulfidptosis-related molecular clusters. (**K**) Boxplots showing the infiltration scores of immune cells among the disulfidptosis-related molecular clusters.

To elucidate the disulfidptosis-related expression patterns in OS, transcriptome samples of OS were grouped based on the expression profiles of 24 DSRGs using a consensus clustering algorithm. The area under the cumulative distribution function (CDF) curves exhibited difference when k = 2–9 (Fig. 2A,B). The number of clusters was most stable when the k value was set to four (k = 4, Fig. 2C). The results of the PCA demonstrated a significant difference among the four clusters (Fig. 2D). As the sample size of cluster D (n = 3) was too small, it was excluded from further analysis.

To explore the different molecular characteristics between clusters, we first comprehensively assessed the differentially expressed genes (DEGs) between the two groups; 24, 18, and 65 DEGs were detected between clusters A and B, clusters B and C, and clusters A and C, respectively, and the three DEG sets were subsequently combined as one main DEG set with 82 genes (Fig. 2E, supplementary file). Gene Ontology (GO) and Kyoto Encyclopedia of Genes and Genomes (KEGG) analyses and according cnet plotting were performed to further explore the functions of the cluster-specific DEGs (SFigure 2). The results indicated that GO functions, including long-chain fatty acid metabolic process, epoxygenase P450 pathway, exogenous drug catabolic process, aromatic amino acid family catabolic process, ion channel complex, transmembrane transporter complex, transporter complex, cation channel complex, gated channel activity, and oxygen binding, were enriched (Fig. 2F,H). KEGG functions including platinum drug resistance, bladder cancer, apoptosis, p53 signaling pathway, pancreatic cancer, Hepatitis B, ErbB signaling pathway, Apoptosis, IL-17 signaling pathway, and endocrine resistance were enriched (Fig. 2G,I). Moreover, the immune ion analysis results showed that an altered immune microenvironment existed among the three clusters; specifically, immune cells including resting CD4 T memory cells, activated CD4 memory T cells, monocytes, and resting dendritic cells were screened as significantly differently infiltrated types of cells (Fig. 2J–K).

### Construction of DSRGs-based OS prognostic model

**Figure 3:**
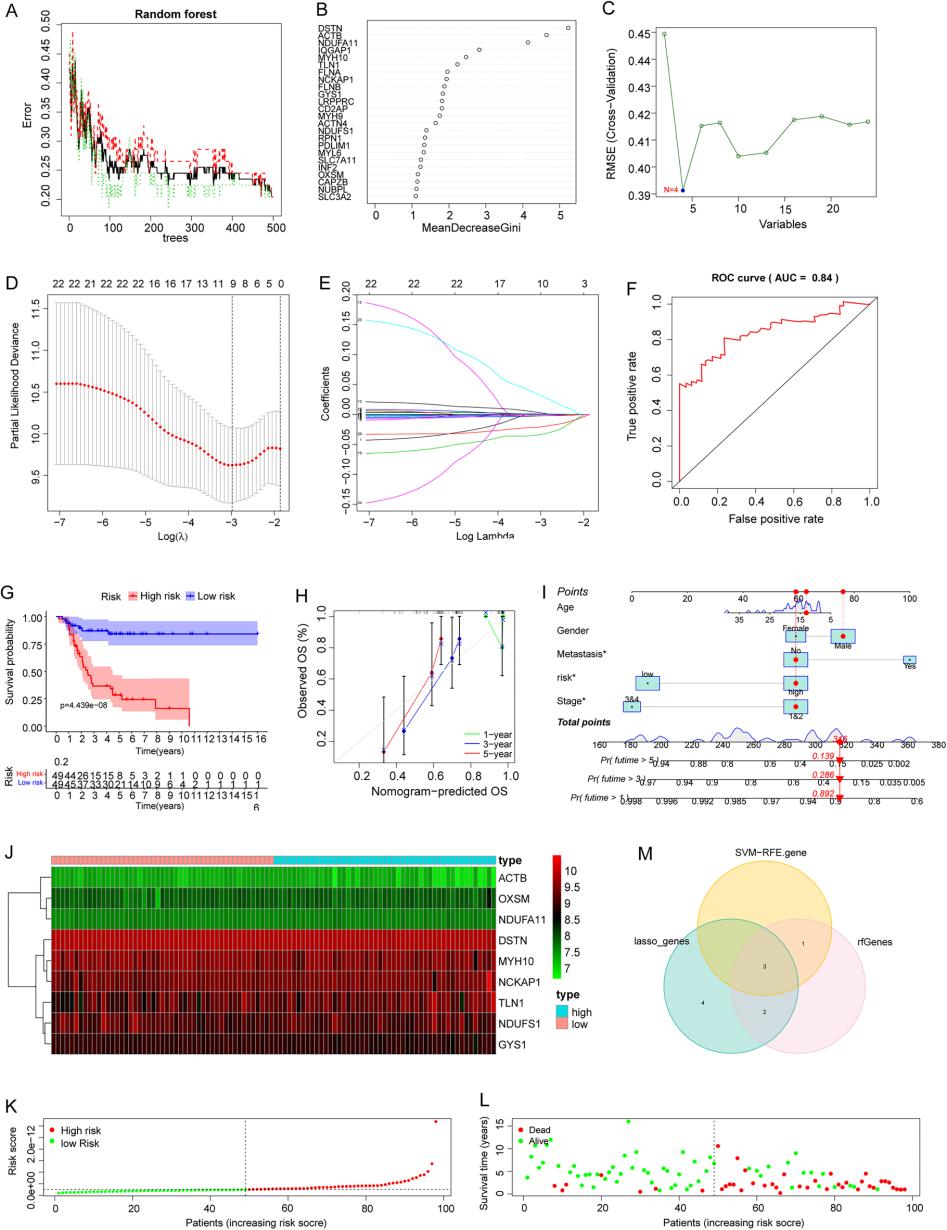
Construction and evaluation of RF, SVM, and LASSO machine models. (**A**,**B**) Error graph of the random forest (RF) models, the genes are screened as meanDecreaseGini increases. (**C**) Support vector machine (SVM) models, the gene number of 4 is decided as RMSE (cross-validation) went the lowest. (**D**) Coefficients in the LASSO regression. (**E**) Cross-validation for tuning parameter selection in the proportional hazards model, the gene number of 9 is decided as Partial likelihood deviance went the lowest. (**F**) ROC analysis of LASSO models. (**G**,**H**) Survival analysis of different groups assessing the independence of the signatures. (**I**) Nomogram for predicting the proportion of patients. (**J**–**L**) The expression of prognosis-related DSRGs, risk score, and patients’ survival status between the high and low risk groups, the shade of the color illustrates the expression value of the genes. (**M**) VENN plot showing the intersection of genes screened by three machine models.

To identify the high diagnostic value of DSRGs, three proven machine learning models, the random forest model (RF), support vector machine model (SVM), and LASSO regression method, were utilized based on the expression profiles of the OS cohort of 98 samples. We constructed the RF prediction model that included *DSTN, ACTB, NDUFA11, IQGAP1, MYH10,* and *TLN1* as six DSRGs (Fig. 3A), and the SVM prediction model included the DSRGs *DSTN, ACTB, NDUFA11*, and *IQGAP1* (Fig. 3B,C). Moreover, the LASSO prediction model included nine DSRGs, namely *NDUFS1, TLN1, MYH1, OXSM, GYS1, DSTN, NCKAP, ACTB,* and *NDUFA11* (Fig. 3D,E). We evaluated the discriminative performance of the LASSO algorithms in the testing set by calculating receiver operating characteristic (ROC) curves (Fig. 3F). To further assess the predictive efficiency of the LASSO model, we constructed survival analysis (Fig. 3G,H) and a nomogram to estimate the risk of OS (Fig. 3I). The expression of prognosis-related DSRGs, risk score, and patients’ survival status between the high and low risk groups were further displayed (Fig. 3J–L). We identified key genes, including *NDUFA11, DSTN*, and *ACTB*, by intersecting the gene sets in the three prognostic models (Fig. 3M).

### Identification and functional diversity of OS subclusters

**Figure 4:**
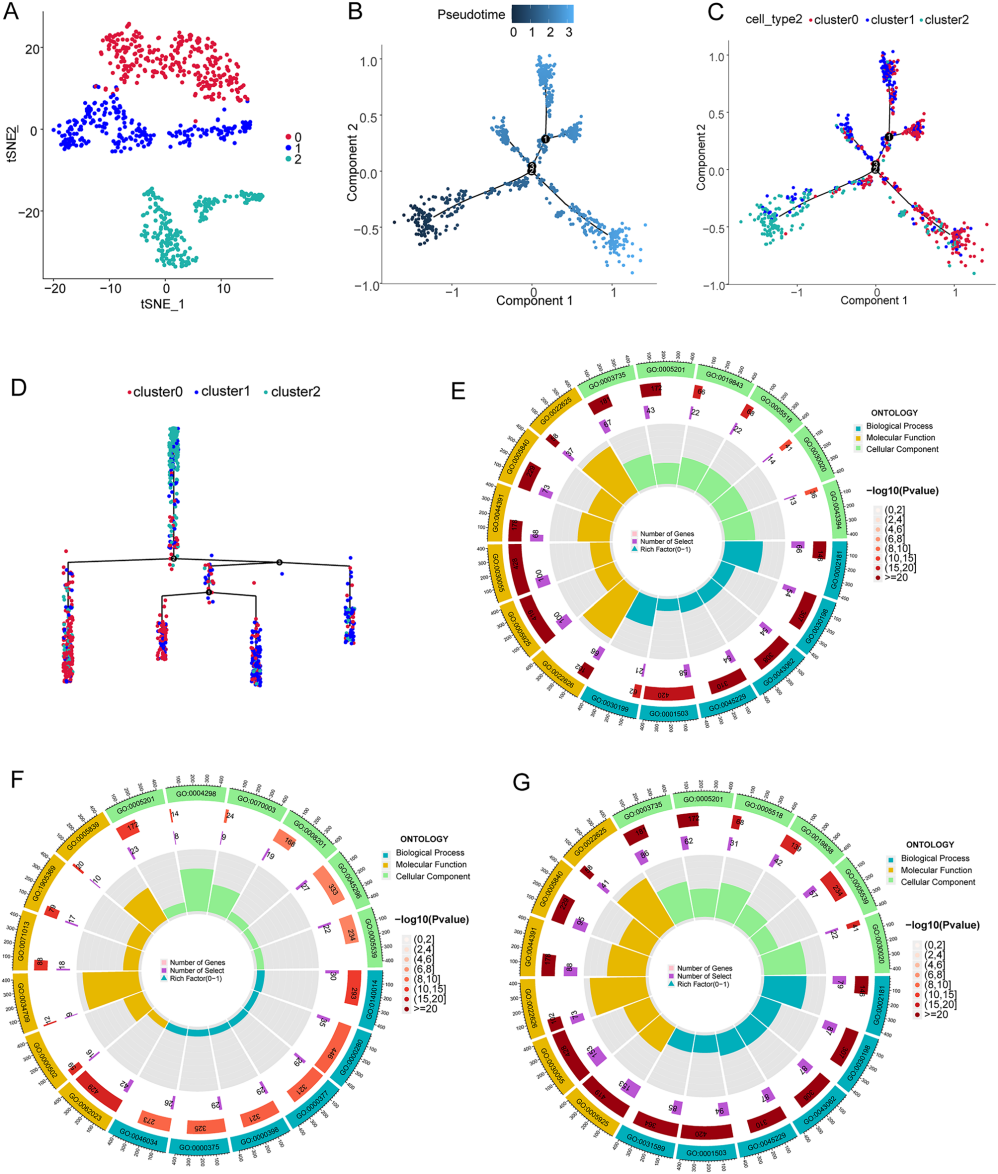
Subgrouping OS cells. (**A**) The t-SNE plot showing the three subclusters of osteosarcoma cells. (**B**) Pseudotime trajectory scatter plot showing pseudotime state of OS subclusters. (**C**,**D**) Pseudotime trajectory scatter plot showing cell types of OS subclusters in the shape of the pseudotime state. (**E**) Circle plot showing the GO enrichment function of cell markers in OS cluster1, different color represent each category of the ontology, (**F**) circle plot showing the GO enrichment function of cell markers in OS cluster2, (**G**) circle plot showing the GO enrichment function of cell markers in OS cluster3.

**Figure 5:**
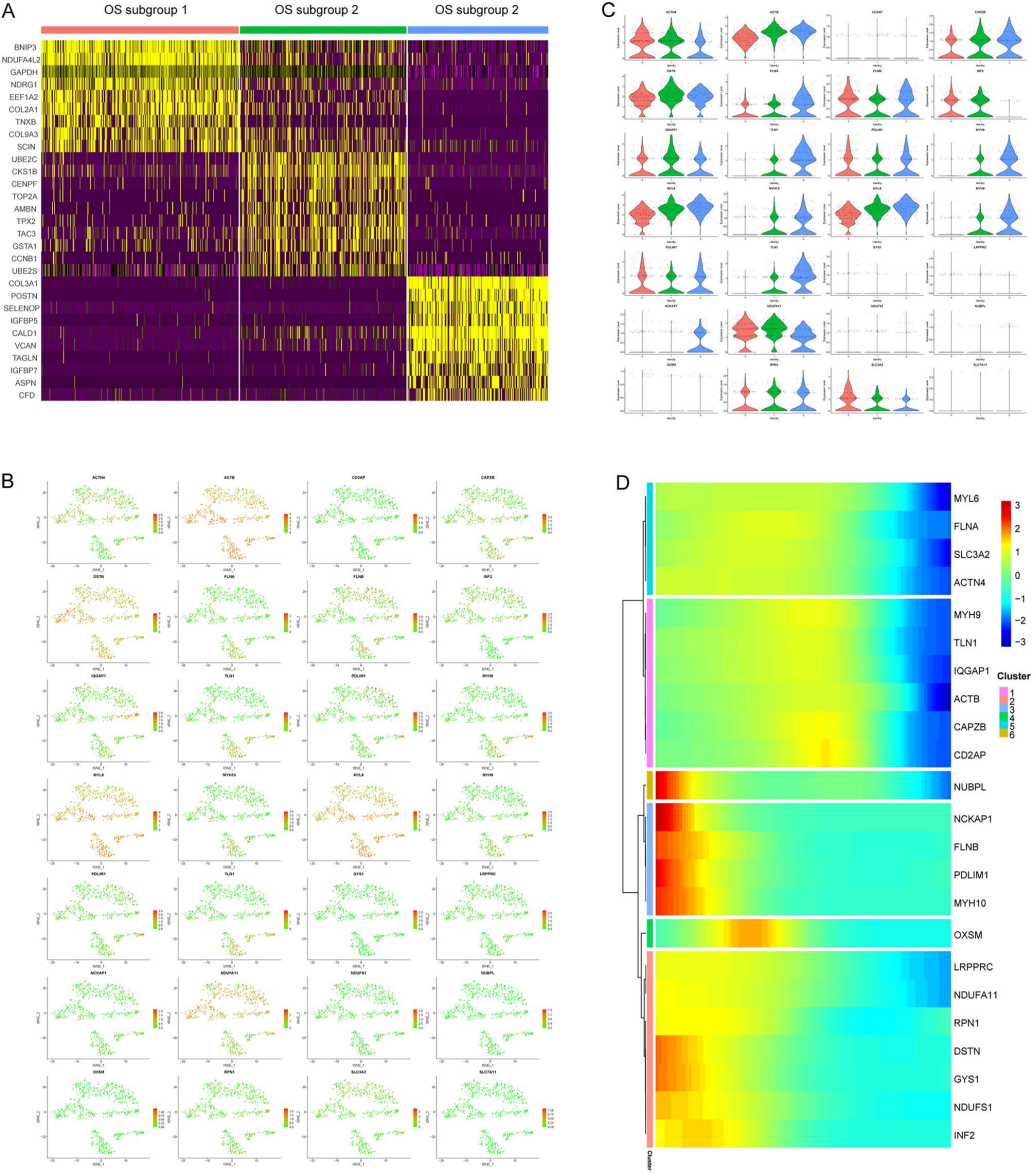
DSRGs expression profiles of OS subclusters in cell components of the OS TME*.* (**A**) Heatmap shows the top differentially expression genes in each OS cluster. (**B**) t-SNE scatter plot showing the expression level of each DSRG in OS subclusters. (**C**) Violin plot showing the expression level of DSRGs in OS subclusters. (**D**) Heatmap shows expression patterns of DSRGs in a pseudotime trajectory profile, the genes are classified into 6 clusters according to the expression pattern.

The OS cells were further divided into three subgroups in order to investigate the disulfidptosis heterogeneity of OS cells (Fig. 4A), the pseudotime trajectory analysis showed that the OS subgroup 2 is located at the start of the cell differentiation (Fig. 4B–D). The marker genes expression heatmap showed a high heterogeneity of gene expression profile (Fig. 5A). Combined with the expression of cell markers and the result of singleR, we found that enriched functions of OS subgroup 1 included the IL-18 signaling pathway, response to oxygen levels, response to hormone, and positive regulation of cell death; thus, we defined them as immune regulation-related OS cells (Fig. 4E). The enriched functions of OS subgroup 2 included extracellular matrix organization, regulation of mitotic cell cycle, and ossification; thus, we defined them as extracellular matrix regulation-related OS cells (Fig. 4F). The enriched functions of OS subgroup 3 included response to hormone, VEGFA-VEGFR2 signaling pathway, and response to growth factor (Fig. 4G); thus, we defined them as angiogenesis regulation-related OS cells. The expression profiles of DSRGs in OS subclusters also illustrate highly heterogeneity, to be specific, the expression of *ACTB* was relatively low in OS subgroup 1, *DSTN* was relatively low in OS subgroup 1, and *NDUFA11* was relatively low in OS subgroup 3 (Fig. 5B,C). The subsequent pseudotime trajectory analysis (Fig. 4G,H) showed that the expression patterns of DSRGs can be divided into six different clusters; for example, DSRGs including *MYL6, FLNA, SLC3A2, ACTN4, MYH9, TLN1, IQGAP1, ACTB, CAPZB,* and *CD2AP* were highly expressed in the early stage of OS differentiation, whereas expression of *NUBPL, NCKAP1, FLNB, PDLIM1*, and *MYH10* was relative low in the early stage of OS differentiation (Fig. 5D).

### DSRGs targeted-intercellular crosstalk in OS

**Figure 6:**
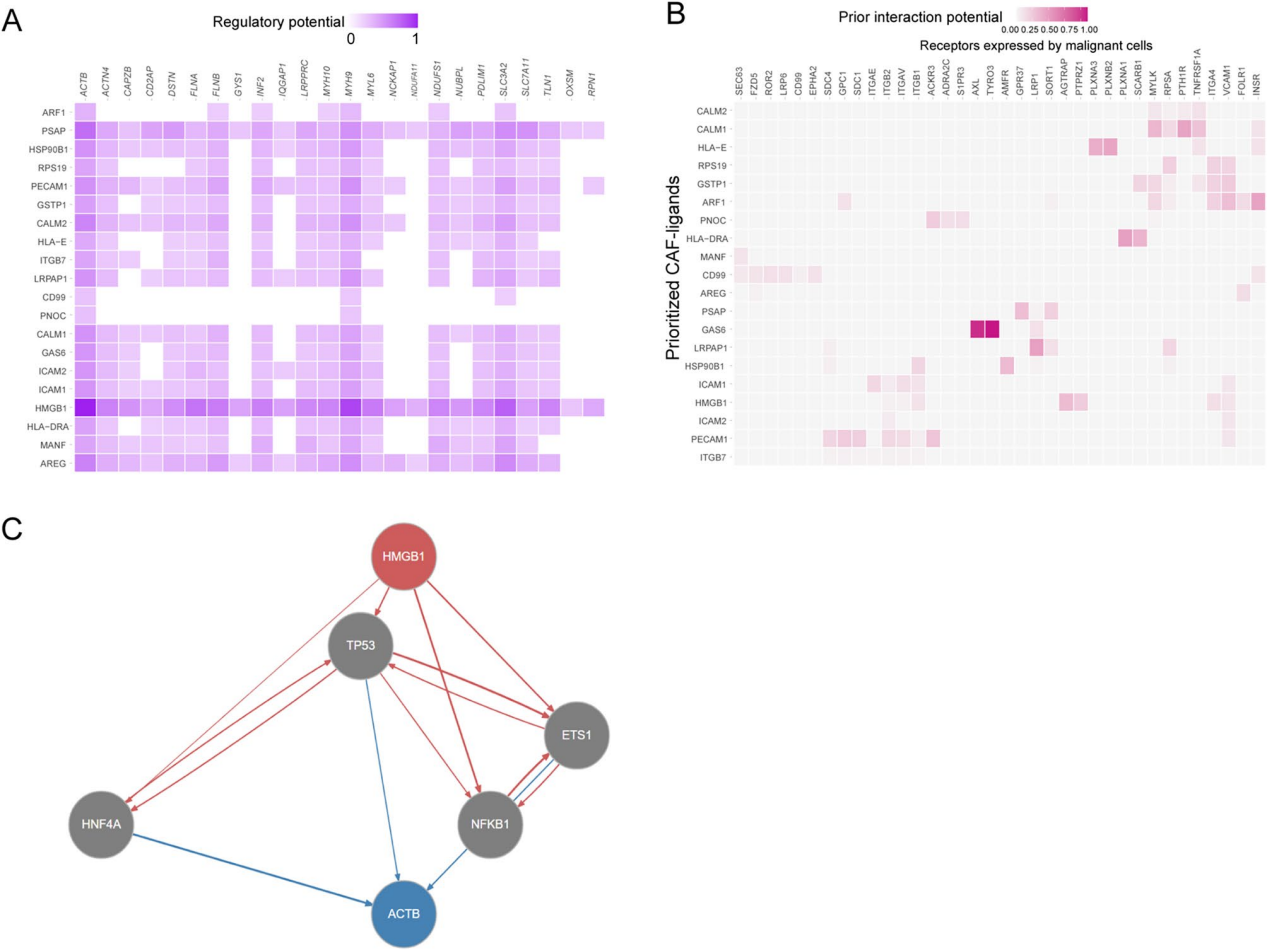
DSRGs-related intercellular communication analysis between OS cells and the niche cells in OS TME*.* (**A**) Heatmap of ligand–target DSRGs interaction in the niche cells-OS cells communication, the darker of the color indicate the higher regulation potential of the ligand to the gene. (**B**) Top ligand–receptor interaction pairs in the niche cells-OS cells communication, the darker of the color indicate the higher interaction potential of the ligand to the gene. (**C**) Predicted signaling pathways of HMGB1-ACTB axis.

To examine DSRGs-related intercellular crosstalk between OS cells and niche cells in OS TME, NicheNet was used to specifically predict the ligand-receptor pairs that potentially regulate transcriptome expression of DSRGs in OS. We first identified the top 20 ligands in the OS TME that potentially affect the expression of DSRGs; interestingly, the ligand HMGB1 had the most profound impact on most of the DSRGs, and the expression of *ACTB, MYH9*, and *SLC3A2* among other DSRGs seemed to be affected the most (Fig. 6A). We also screened the top 20 ligands that affected OS cells and confirmed the presence of HMGB1 (Fig. 6B). Next, we predicted the signaling pathway from HMGB1 to DSRGs, which showed that TP53, ETS1, NFKB1, and HNF4A were involved in the regulation of HMGB1 to ACTB axis, this provided an insight into the molecular mechanism of the exogenous regulation of disulfidptosis (Fig. 6C, SFigure 4A–M).

### Screening and characterization of DSRGs hub genes

**Figure 7:**
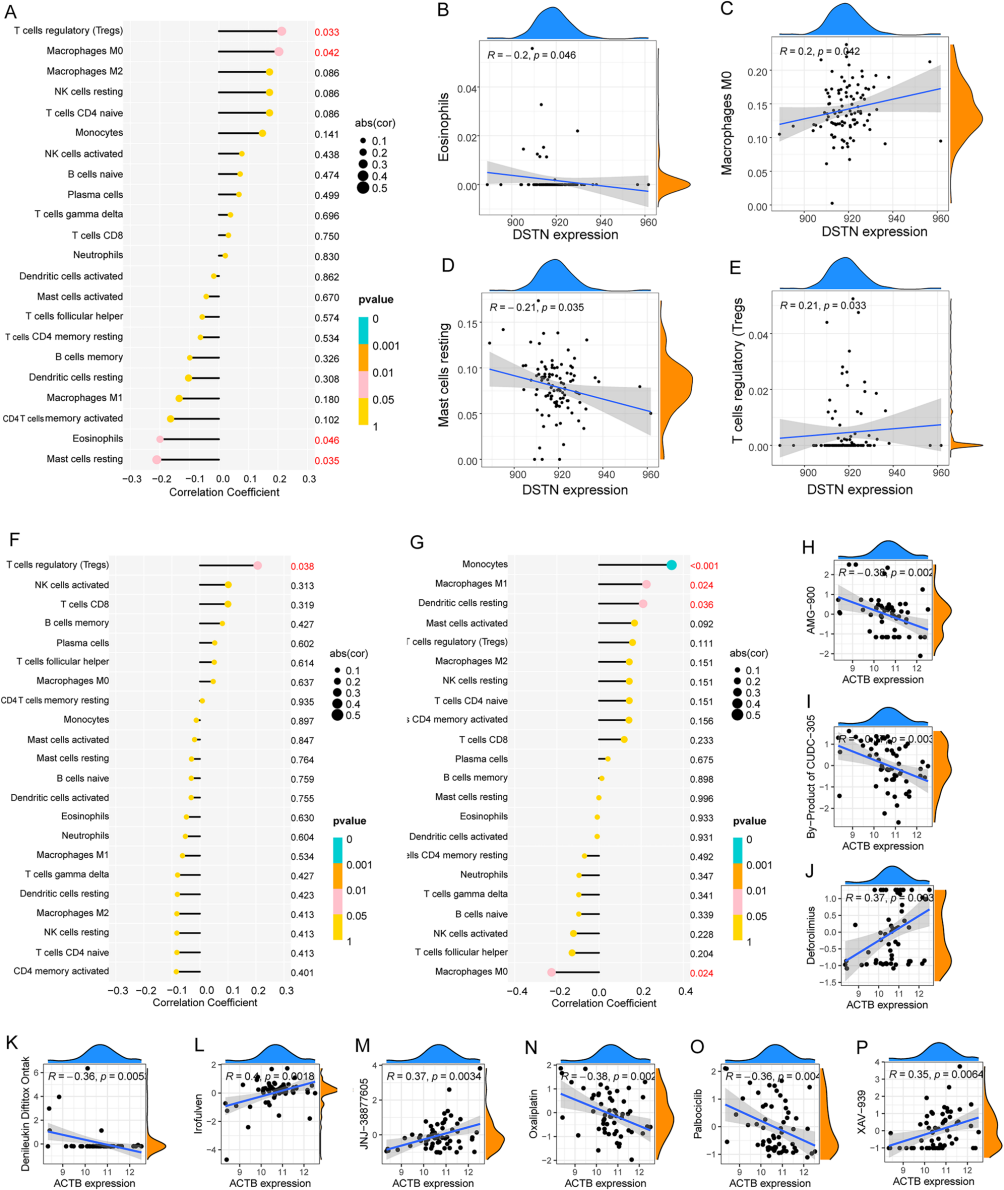
immune and drug sensitive analysis of hub genes. (**A**–**G**) Correlation analysis between ACTB and infiltration scores of immune cells, the red ones indicate significant differences. (**H**–**P**) Correlation analysis between ACTB and different drug sensitive scores, the positive number of R shows the positive correlation and the negative number of R shows the negative correlation.

**Figure 8:**
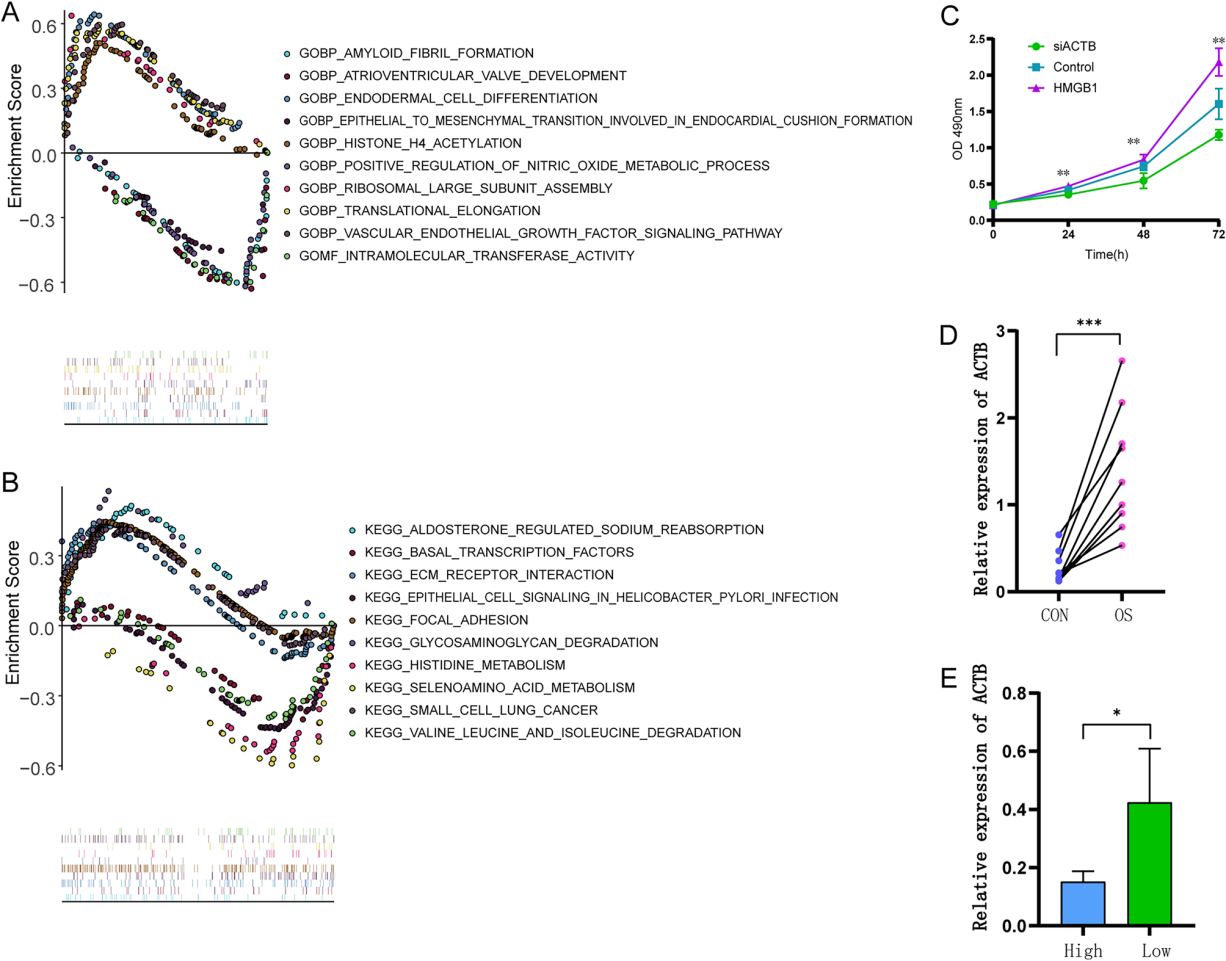
GSEA analysis of ACTB and in vitro experiment. (**A**) GSEA-GO analysis of ACTB, (**B**) GSEA-KEGG analysis of ACTB. (**C**) 143B cell viability after treatment with siACTB, detected by a cell growth curve plotting experiment. (**D**) The *ACTB* expression level in OS and normal tissue. (**E**) *ACTB* expression levels in higher and lower immunity groups. “High” represents the higher immunity group and “low” represents the lower immunity group.

We validated the correlation between hub genes and infiltration of immune cells, which revealed that the expression of *NDUFA11* was positively correlated with resting dendritic cells, M1 macrophages, and monocytes and negatively correlated with M0 macrophages (Fig. 7A–E). The expression of *ACTB* was positively correlated with M0 macrophages and T regulatory cells (Tregs) and negatively correlated with resting mast cells and eosinophils (Fig. 7F). The expression of *DSTN* was positively correlated with Tregs (Fig. 7G). Furthermore, the correlation between the hub genes and the potential drugs were validated. AMG-900, CUDC-305, deforolimus, denileukin diftitox (Ontak), irofulven, JNJ-38877605, oxaliplatin, palbociclib, and XAV-939 were screened as potential drugs that target ACTB in OS (Fig. 7H–P). We then performed a differential functional annotation analysis between ACTB-high expression groups and ACTB-low expression groups of transcriptome data. The top GSEA-GO enrichment analysis results included amyloid fibril formation, atrioventricular valve development, endodermal cell differentiation, epithelial to mesenchymal transition involved in endocardial cushion formation, histone h4 acetylation, positive regulation of nitric oxide metabolic process, ribosomal large subunit assembly, translational elongation, vascular endothelial growth factor signaling pathway, and intramolecular transferase activity (Fig. 8A). The top GSEA-KEGG enrichment analysis results included aldosterone-regulated sodium reabsorption; basal transcription factors; Ecm receptor interaction; epithelial cell signaling in H. pylori infection; focal adhesion; glycosaminoglycan degradation; histidine metabolism; selenoamino acid metabolism; small cell lung cancer; and valine, leucine, and isoleucine degradation (Fig. 8B).

### In vitro siACTB or HMGB1 treatment regulates survival of OS cells

To determine whether *ACTB* expression and exogenous HMGB1 are involved in the survival of OS cells, si*ACTB* and exogenous HMGB1 were separately applied to the culture system of 143B cells. The western blot result showed that the expression of *ACTB* significantly decreased after si*ACTB* treatment (with P value of 0.0075, SFigure 3). Cell activity was observed by plotting cell growth curve, the result showed that tumor cell viability of 143B cells decreased after siACTB treatment and increased after HMGB1 treatment (Fig. 8C).

### Higher expression of ACTB in tumors from patients with low immunity scores

The expression levels of *ACTB* in each patient were determined using qRT-PCR. The expression of *ACTB* in OS tissue (nine cases) and the low immunity score group (four cases) was significantly higher than that in the adjacent tissue (nine cases) and the higher immunity score group (five cases, Fig. 8D,E).

## Discussion

Chemical resistance is one of the main obstacles in OS treatment. Therefore, it is important to identify methods to effectively induce cell death and overcome chemical resistance during OS treatment. Various types of cell death, including programmed and unprogrammed^22^, play important roles in the development and killing of tumors^23^. In recent years, the concept of programmed cell death has expanded, which includes apoptosis, autophagy, necrotic apoptosis, and others^24–26^. Recent studies have suggested a potential relationship between ferroptosis and malignant growth in OS; according to Isani et al., OS cell lines show ferroptosis with iron-dependent and non-apoptotic features^27^. Ferroptosis sensitization can be induced by inhibiting STAT3/Nrf2/GPx4 signaling, which enhances the sensitivity of OS cells to cisplatin^28^. Reportedly, necroptosis signaling pathway also contributes in the chemotherapy treatment of OS, and the anti-tumor effects of shikonin on OS were partly due to induction of RIP1 and RIP3 dependent necroptosis; indicating that shikonin could be a potential anti-tumor agent for the treatment of primary and metastatic OS^29^. Two compounds (doxorubicin and staurosporine) selectively target OS cells, induce caspase 3 and 7 activity in U3OS cells, and promote apoptosis in OS cell line^30^. In addition, various cell death-related gene signatures have been constructed to predict the prognosis of patients with OS, including proptosis-related lncRNA-based and anoiki-based prognostic signatures^31,32^.

Identification and characterization of regulatory cell death mechanisms could enhance our understanding of cell homeostasis in physiological states and provide insights into the treatment of many diseases, including cancer^33^. Liu et al. suggested that actin-cytoskeletal proteins can be affected by disulfide stress caused by the excessive accumulation of disulfide molecules in cells, resulting in the formation of abnormal disulfide bonds between actin-cytoskeletal proteins, which if left unrepaired, lead to the breakdown of the actin network and ultimately lead to a novel cell death known as disulfidptosis. However, in cells with high SLC7A11 expression and glucose starvation, homocysteine uptake and insufficient NADPH supply lead to NADPH depletion. This combination induces abnormal disulfide binding in actin cytoskeletal proteins, ultimately leading to actin network breakdown and subsequent disulfidptosis^11^. Thus, GLUT suppression-induced disulfidptosis may be an effective treatment strategy for SLC7A11 high-expression tumor cells^14^. Our results further uncovered the disulfidptosis feature of OS, which has been characterized by high SLC7A11 expression^15^. Inhibition of SLC7A11 reduces glutathione production, thereby promoting OS cell death and inhibiting OS growth and metastasis^34^. We then detected its potential roles in both TME and OS subcluster and found a relatively high disulfidptosis level in OS cells, TAMs, and endothelial cells. TAMs are crucial components involved in tumor immunity and affect the efficacy of tumor immunotherapy through their polarization. Previous studies have shown that inhibiting ferroptosis in tumors can regulate the immunosuppressive phenotype of TAMs. In addition, we found that multiple DSRGs, including *ACTB* and *MYL6*, regulate the cell cycle-specific expression features. It has been reported that the cytoskeleton system is highly involved in the regulation of the cell cycle^35,36^. Our study revealed a potentially important role of the cell cycle in disulfidptosis.

Cell death may activate the tumor immunity through releasing dead cell antigens, which further leads to “immunogenic cell death” (ICD)^37^. For example, several cytotoxic antineoplastics stimulate tumor immunity by inducing cancer cell death^38^. Our correlation analysis results indicated that immune cells, including resting CD4+ memory T cells, activated CD4+ memory T cells, monocytes, and resting dendritic cells, may play important roles in ICD after OS disulfidptosis,. Studies have shown that OS could reduce the proliferation rate of T cells, increase the regulatory (FoxP3+) CD4+ phenotype, and reduce the expression of the activation marker CD8+ on CD25+ cells through multiple approaches^39^. Interestingly, our intercellular communication analysis indicated a crucial role of HMGB1 in communication-induced disulfidptosis-related gene expression regulation. HMGB1 protein is usually present in the nucleus and is translocated to the cytoplasm for cell death-associated release; it acts as a controlled universal DAMP that regulates ICD through its abundance and oxidation status^40^, and extracellular HMGB1 assists the induction of immune tolerance in antigen-presenting cells and promotes the expression of immune checkpoint molecules^41,42^. Thus, our results indicated a crucial function of HMGB1 in disulfidptosis-induced ICD.

We further screened three genes related to OS disulfide death, including *NDUFA11, DSTN,* and *ACTB*, by combining the results of multiple machine learning algorithms. *NDUFA11* encodes a subunit of membrane-bound mitochondrial complex I. Inhibition of complex I activity has been reported to enhance ROS production and promote cancer cell migration and invasion^43^. Complex I subunits play a key role in ROS generation. Studies have proven that silencing of *NDUFA11* inhibits superoxide and ROS production in breast cancer cells. Furthermore, upregulation of complex I subunits in RORα-silenced mammary epithelial cells is accompanied by higher oxygen consumption rate, indicating that increased expression of NDUFA11 in mammary epithelial cells enhances electron leakage in mitochondria^44^. DSTN belongs to the actin-binding protein (ADF)/cofilin family of proteins. This family of proteins is responsible for enhancing the turnover rate of actin in vivo^45^ and participates in multiple cellular biological processes, such as cell division, proliferation, and membrane trafficking^46,47^. Studies have shown that actin dynamics and its regulation of target proteins are critical to the development and function of healthy cells. As a member of the ADF/cofilin protein family, relatively few studies have focused on DSTN, which have reported that DSTN expression affects the migration and invasion of colon cancer^48^. High expression of DSTN is also associated with pancreatic cancer growth and perineural invasion^49^. Recent studies have shown that overexpressed DSTN can upregulate the activity of Wnt/β-catenin signaling pathway by promoting the nuclear translocation of β-catenin and the interaction between β-catenin and TCF4, thereby promoting tumor proliferation and metastasis^50^. We then focused on *ACTB*, which encodes an actin protein involved in cell motility, structure, integrity, and intercellular signaling. ACTB upregulation in cancer may regulate tumor cell proliferation, phenotype, and metastasis, thereby further affecting tumor malignancy and prognosis in patients with tumor. Compared with that in the normal tissues, the expression of ACTB is increased in head and neck cancer, leukemia, pancreatic cancer, and other cancers. Moreover, the expression of ACTB in stage III and IV of various cancers is higher than that in stage I and II^51^. Our study has shown that ACTB may play a specific role in regulating the immune microenvironment of OS by revealing a significant correlation between ACTB expression and the level of infiltration of M0 macrophages, Tregs, resting mast cells, and eosinophils cells. Previous studies have shown that ACTB expression exerts a synergistic effect on immune checkpoint members and other immune modulators in various cancers^51^. Consistent with previous studies, our results suggest that ACTB is a potential target for immunotherapy. Subsequently, we performed a single-gene functional enrichment analysis of hub genes, including *ACTB*, in OS and revealed that ACTB has multiple functions that are potentially related to the mechanism of cancer development, including epithelial-to-mesenchymal transition involved in endocardial cushion formation. We further screened for drugs that could potentially target ACTB in OS. Oxaliplatin (OXA) is a widely used clinical antineoplastic drug. OXA can activate anti-tumor immunity by inducing ICD of tumor cells, and can also induce the accumulation of cytotoxic T lymphocytes^52^. Palbociclib, as a cyclin-dependent kinase (CDK) inhibitor, can inhibit the growth of patient-derived orthotopic xenograft (PDOX) in OS, Ewing sarcoma, dedifferentiated liposarcoma, and peritoneal metastatic leiomyosarcoma significantly^53^. However, the targeting effect of these drugs against ACTB remains unclear. Thus, the relevant mechanisms need to be further explored to develop new combination therapies against disulfidptosis.

## Conclusions

Our study on disulfidptosis provides key insights for understanding and further targeting this unique cell death type mechanism for the treatment of OS.

### Supplementary Information

Supplementary Information.

Supplementary Figures.

## Data Availability

Restrictions apply to the availability of these data. Single-cell RNA-sequencing data were obtained from the GEO database https://www.ncbi.nlm.nih.gov/geo/query/acc.cgi?acc=GSE152048. OS transcriptome sequencing data were downloaded from the Cancer Genome Atlas (TARGETs) https://ocg.cancer.gov/programs/target.
