# Supplementary figures and images for: Transcriptome and single-cell analysis reveal disulfidptosis-related modification patterns of tumor microenvironment and prognosis in osteosarcoma
Source: Sci Rep. 2024 Apr 22;14:9186. doi: 10.1038/s41598-024-59243-9 (PMC11035678; doi:10.1038/s41598-024-59243-9)

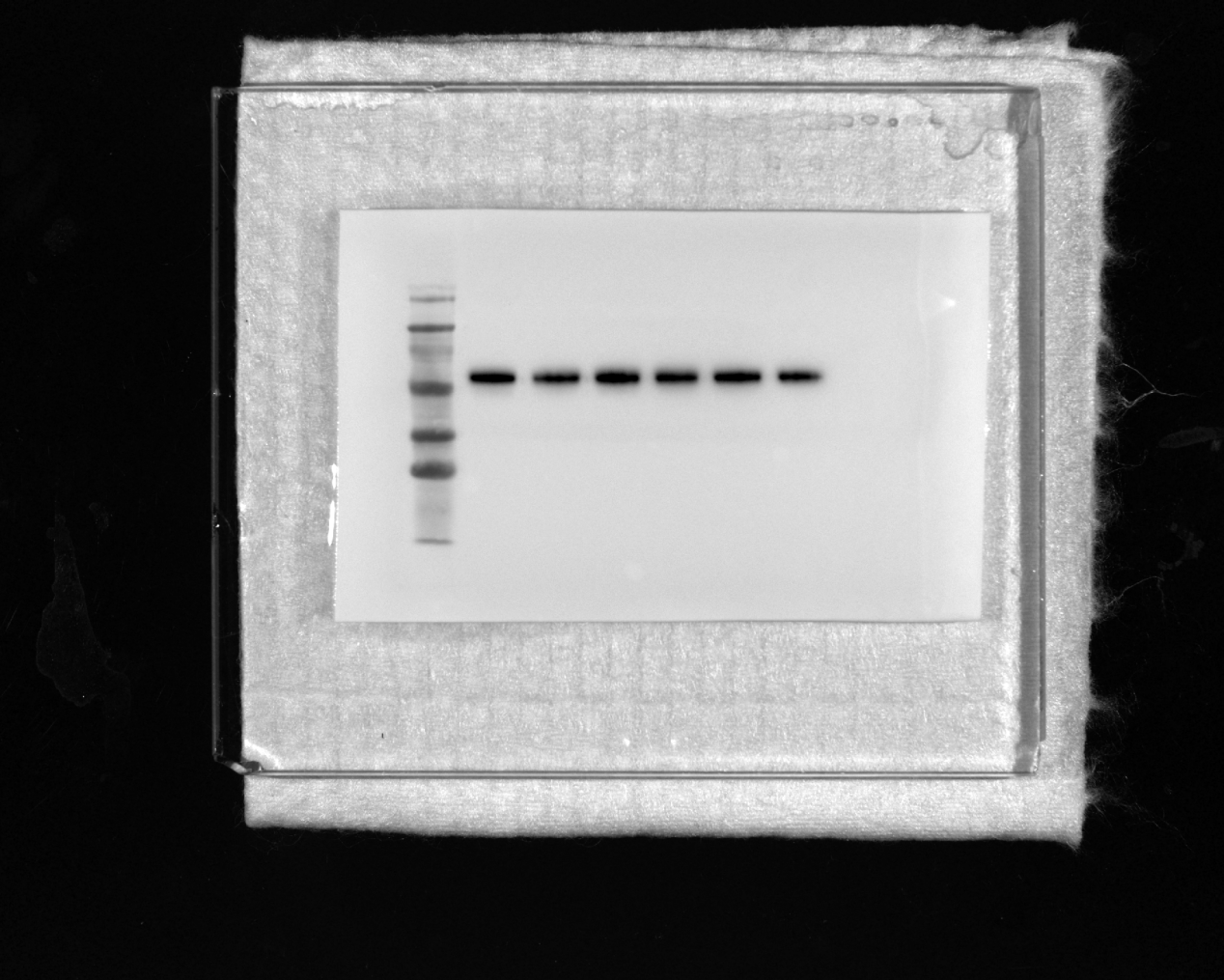


Expression of ACTB


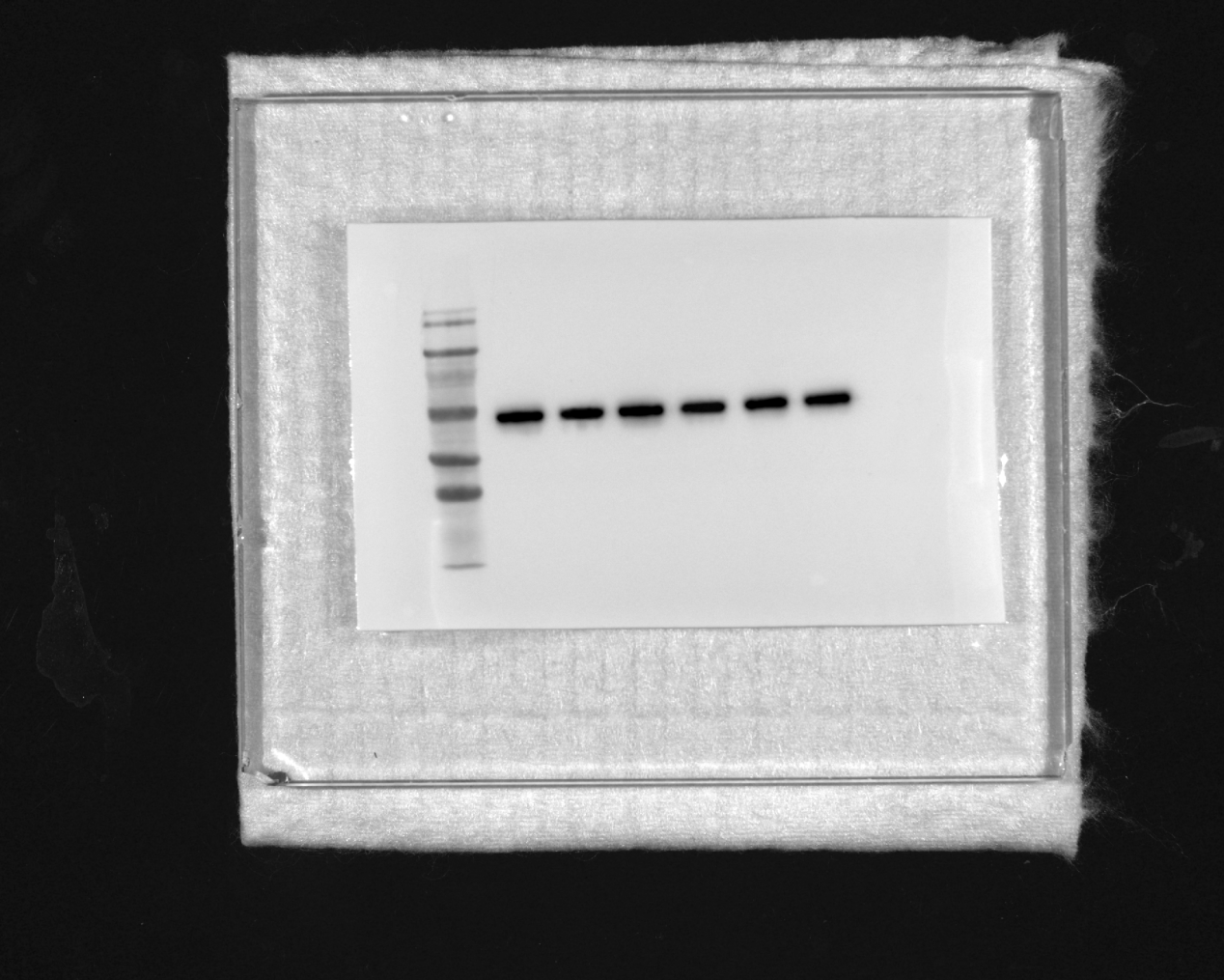


Expression of GAPDH

Supplement: Supplementary file 1 — Supplementary Information. [file 41598_2024_59243_MOESM1_ESM.docx]
