# Supplementary material for: Transcriptome and single-cell analysis reveal disulfidptosis-related modification patterns of tumor microenvironment and prognosis in osteosarcoma
Source: Sci Rep. 2024 Apr 22;14:9186. doi: 10.1038/s41598-024-59243-9 (PMC11035678; doi:10.1038/s41598-024-59243-9)
Supplement: Supplementary file 2 — Supplementary Figures. [file 41598_2024_59243_MOESM2_ESM.docx]

Figure S1 quality control of the single-cell analysis


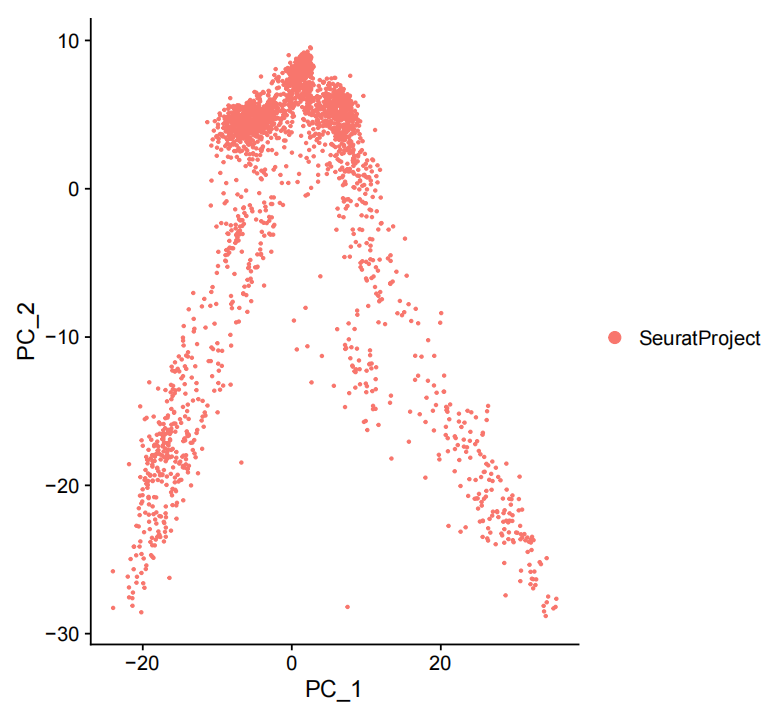


SFigure1 legend

Quality control of single-cell analysis by conducting PCA analysis.

Figure S2 Cnet plotting of the GO analysis.


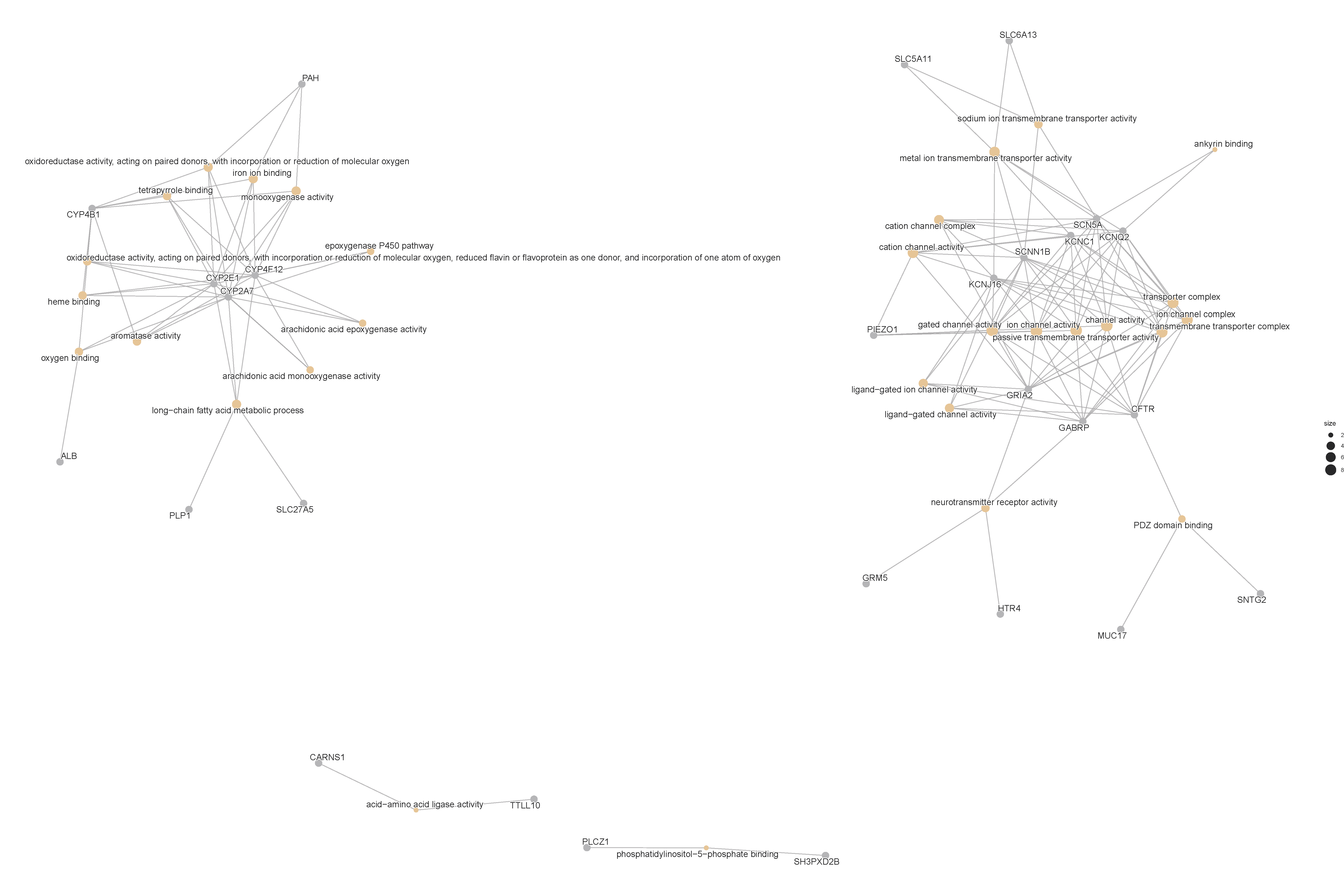


SFigure2 legend

The gene names is shown in color grey and the enriched pathways is shown in color yellow, the size of the circle represents the enrich degree of pathways.

Figure S3 WB experiment.


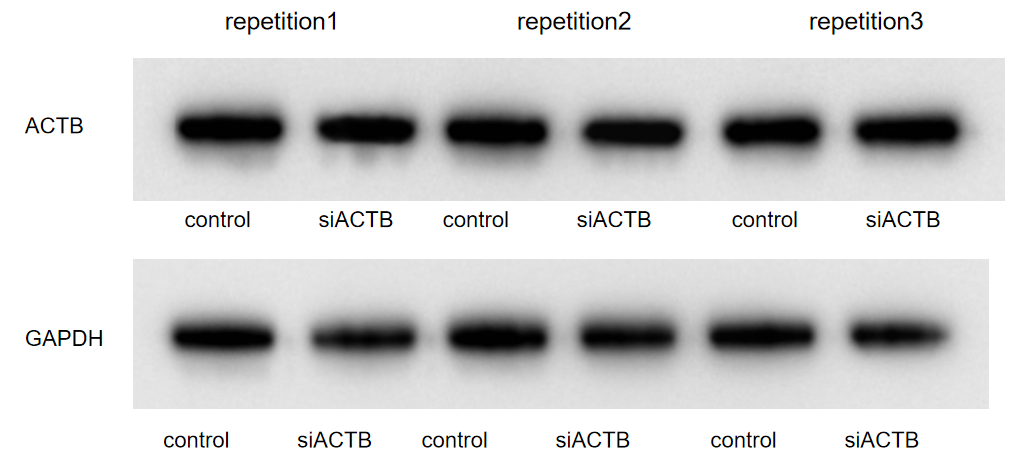

SFigure3 legend

WB experiment of the siACTB knock-down validation.

Figure S4 DSRGs targeted-intercellular crosstalk in OS


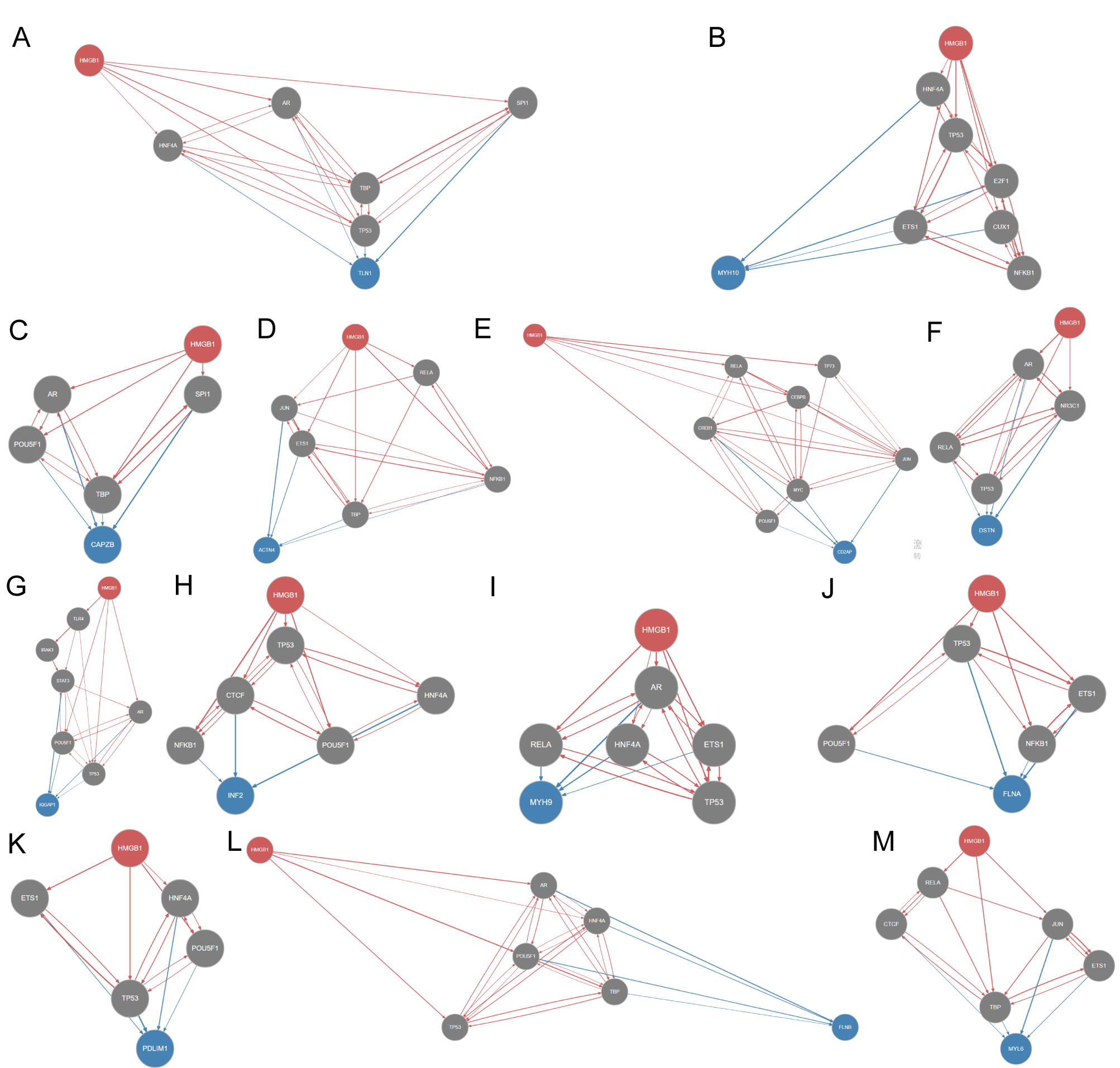


SFigure4 legend

A-M. Predicted signaling pathways of HMGB1-target DSRGs axis.
